# Plug-and-Play Biointerfaces: Harnessing Host–Guest Interactions for Fabrication of Functional Polymeric Coatings

**DOI:** 10.1021/acs.biomac.3c00360

**Published:** 2023-07-05

**Authors:** Aysun Degirmenci, Rana Sanyal, Amitav Sanyal

**Affiliations:** † Department of Chemistry, Bogazici University, Bebek, Istanbul 34342, Türkiye; ‡ Center for Life Sciences and Technologies, Bogazici University, Istanbul 34342, Türkiye

## Abstract

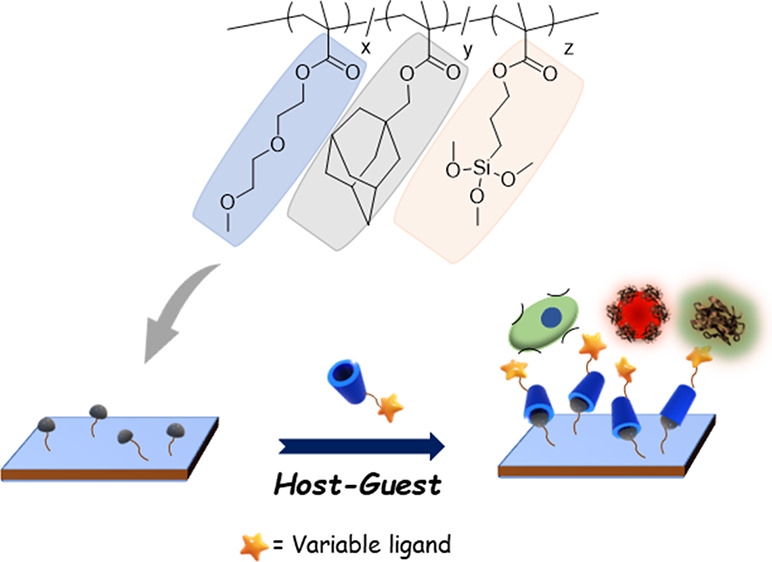

Polymeric surface coatings capable of effectively integrating desired functional molecules and ligands are attractive for fabricating bio-interfaces necessary for various applications. Herein, we report the design of a polymeric platform amenable to such modifications in a modular fashion through host–guest chemistry. Copolymers containing adamantane (Ada) moieties, diethylene glycol (DEG) units, and silyloxy groups to provide functionalization handles, anti-biofouling character, and surface attachment, respectively, were synthesized. These copolymers were employed to modify silicon/glass surfaces to enable their functionalization using beta-cyclodextrin (βCD) containing functional molecules and bioactive ligands. Moreover, surface functionalization could be spatially controlled using a well-established technique like microcontact printing. Efficient and robust functionalization of polymer-coated surfaces was demonstrated by immobilizing a βCD-conjugated fluorescent rhodamine dye through the specific noncovalent binding between Ada and βCD units. Furthermore, biotin, mannose, and cell adhesive peptide-modified βCD were immobilized onto the Ada-containing polymer-coated surfaces to direct noncovalent conjugation of streptavidin, concanavalin A (ConA), and fibroblast cells, respectively. It was demonstrated that the mannose-functionalized coating could selectively bind to the target lectin ConA, and the interface could be regenerated and reused several times. Moreover, the polymeric coating was adaptable for cell attachment and proliferation upon noncovalent modification with cell-adhesive peptides. One can envision that the facile synthesis of the Ada-based copolymers, mild conditions for coating surfaces, and their effective transformation to various functional interfaces in a modular fashion offers an attractive approach to engineering functional interfaces for several biomedical applications.

## Introduction

Over the past decades, functional polymeric coatings on surfaces have become prominent due to their widespread use in biomedical sciences.^1−5^ Applications involving biomolecular immobilization, analyte detection, biosensing, targeted drug delivery, and cell adhesion often require the installation of appropriate functional molecules and ligands onto solid surfaces. Polymeric coatings on solid surfaces offer a versatile interface for enabling the abovementioned applications.^6−15^ Generally, one of the two methods is utilized for incorporating functional molecules on a polymeric interface: covalent conjugation or immobilization of noncovalent interactions. While covalent methods for surface immobilization are robust,^16,17^ noncovalent methods offer flexibility such as facile reversibility and renewability/reusability of functional interfaces.^18−21^ Also, in addition to the aforementioned advantages, noncovalent surface functionalization strategies offer an economical approach to achieve diversity in a modular fashion,^22^ and importantly, the approach can be engineered to be nondestructive to the substrate and the guest molecule.^23^

To date, supramolecular host–guest interactions have been extensively explored for versatile and facile modification of various interfaces. Fabrication methods using host–guest supramolecular interactions have attracted significant attention since self-assembly processes drive the association of building blocks at the molecular level.^24−26^ Among reported macrocyclic host molecules, crown ethers,^27^ cyclodextrins (CDs),^28,29^ calixarenes,^30^ and calixpyrroles^31^ are among the most widely explored ones. In addition to these traditional hosts, cucurbiturils (CB[*n*]),^32^ blue-box,^33^ pillar[*n*]arenes,^34^ and resorcinarene^35^ have attracted attention in recent years. Among these macrocyclic structures, CDs have been widely used to fabricate polymeric self-assembled materials for biomedical applications due to their ready availability, low toxicity, excellent biocompatibility, and ability to make strong complexation with a variety of guests.^36−41^ Among CD-based host–guest pairs, adamantane (Ada) is a rigid hydrophobic molecule known to form a stable complex with βCD with a high association in an aqueous environment.^42^ Therefore, the assembly of βCD and Ada has been extensively used in various areas such as drug and gene delivery, biomolecule immobilization, bio-separation, and fabrication of sensors.^43−49^ Among these applications, the construction of functional polymeric interfaces to enable reversible immobilization of biomolecules has been explored in recent years.^18,50−56^ In surface coatings, Chen and coworkers, in a seminal contribution, fabricated Ada-containing polymer brushes to construct surfaces capable of molecular recognition.^18,50^ They fabricated Ada-containing thermo-responsive polymer brushes and showed tunable and reversible integration of biomolecules onto their platform.^18^ In addition, the same group reported polymer brush-coated surfaces with varying thermo-sensitivity and surface wettability by adjusting the amount of the Ada moiety on the polymer brush.^51^ While polymer brushes offer fine-tuned interfaces, their fabrication on surfaces often involves multiple steps, such as initiator immobilization and polymerization under controlled environments. A simple approach involving coating the surface by incubation in a solution containing the polymer will offer a practical alternative. The latter approach will also benefit from the relative ease in chemical characterization of the polymeric component before coating, compared to the characterization of surface-tethered polymers. Hence, one can envision that a polymeric surface coating capable of undergoing a modular plug-and-play integration of functional motifs such as bioactive ligands would be an attractive approach for engineering interfaces for various biomedical and biotechnological applications.

**Scheme 1:**
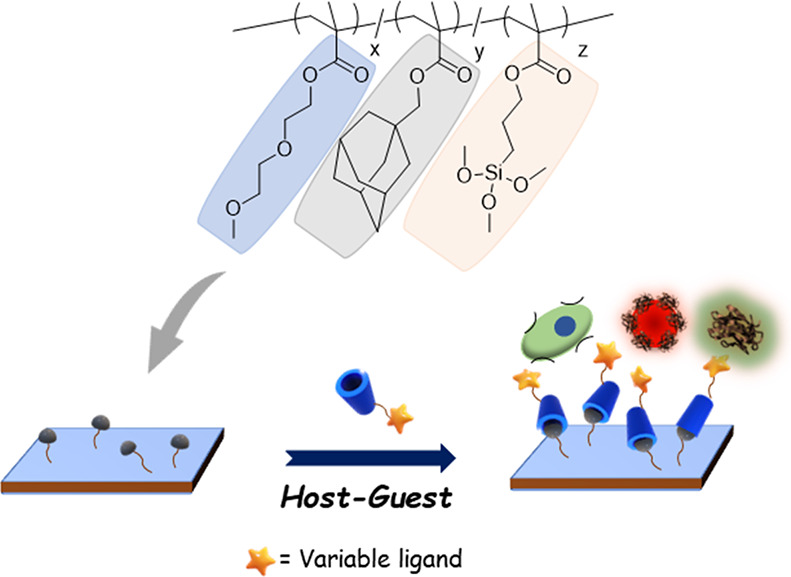
Schematic Illustration of the Fabrication of Polymer-Coated Surface and Subsequent Modification through Host–Guest Chemistry

Herein, we report the fabrication of an adamantane-containing polymeric coating to modify silicon/glass surfaces and render them functionalizable in a modular fashion using CD-based host–guest interactions (Scheme 1). In particular, a surface-reactive copolymer was synthesized using an Ada-containing methacrylate (AdaMA), di(ethylene glycol) based methacrylate (DEGMEMA), and 3-(trimethoxysilyl)propyl methacrylate (TMSPMA) monomers. While the Ada-based monomer is used for inducing host–guest interaction-based specific functionalization, the ethylene glycol-based monomer would impart the necessary anti-biofouling characteristic to the coating. Incorporation of the alkoxysilyl monomer enables robust anchoring of the copolymer onto the glass interfaces. Facile surface functionalization using a variety of functional molecules such as fluorescent dyes, bioactive ligands, and cell adhesive peptides is demonstrated to highlight the versatile nature of the polymeric coating. Furthermore, using microcontact printing,^57^ functionalizations at the interfaces are achieved in a spatially controlled manner, as demonstrated through many of the reported functionalizations.

## Experimental Section

### Materials

All solvents were purchased from Merck and used as obtained without further purification. Ultrapure water was obtained from a Milli-Q Water Purification System (Milli-Q system, Millipore, Billerica, MA, USA). Anhydrous toluene, tetrahydrofuran (THF), and dichloromethane (CH_2_Cl_2_) were obtained from the SciMatCo purification system, and other solvents were dried over molecular sieves. Di(ethylene glycol) methyl ether methacrylate (DEGMEMA) was obtained from Sigma-Aldrich and used after filtration through the activated aluminum oxide. 2,2′-Azobis(2-methylpropionitrile) (AIBN) was purchased from Sigma Aldrich and recrystallized from methanol before use. 3-(Trimethoxysilyl)propyl methacrylate (TMSPMA), 4-cyano-4-(thiobenzoylthio)pentanoic acid, 1-adamantanemethanol, beta-cyclodextrin (βCD), *N*,*N*,*N*′, *N*,″*N*″-pentamethyldiethylenetriamine (PMDETA), copper(I) bromide (CuBr), FITC-streptavidin conjugate, and 4′,6-diamidino-2-phenylindole (DAPI) were purchased from Sigma-Aldrich. Diisopropylethylamine (DIPEA) was obtained from Fluka. Concanavalin A (FITC conjugate) was purchased from Thermo Fisher. CdSeQdot 605-streptavidin conjugate and Alexa Fluor 488 phalloidin were purchased from Invitrogen. Rhodamine-PNA conjugate was purchased from Vectorlabs. d-Mannose and d-(+)-biotin and methacryloyl chloride were obtained from Alfa-Aesar. Cyclo (arg-gly-asp-d-phe-lys-(mal)) was purchased from Peptides International. Lissamine rhodamine B sulfonyl chloride was purchased from Acros Organics. AdaMA monomer,^58^ alkyne-bearing d-mannose,^58^ 6-*O*-monotosyl-βCD (βCD-OTs),^59^ 6-monodeoxy-6-monoazido-βCD (βCD-N_3_),^59^ 6-monodeoxy-6-monoamino-βCD (βCD-NH_2_),^59^ NHS-activated biotin (Biotin-NHS),^60^ βCD-biotin,^60^ βCD-mannose,^61^ and 6-monodeoxy-6-monothio-βCD (βCD-SH)^62^ were synthesized according to the literature procedures.

### Characterization and Methods

^1^H and ^13^C NMR spectra were recorded on Bruker Avance Ultrashield 400 (400 MHz). Molecular weights of the synthesized polymers were estimated by size exclusion chromatography (SEC) using a PSS-SDV (length/ID 8 × 300 mm, 10 μm particle size) gram linear column calibrated with poly(methyl methacrylate) (PMMA) standards using a refractive-index detector with a mobile phase solution of 0.05 M lithium bromide in DMAc as the eluent at a flow rate of 1 mL/min at 30 °C. Attenuated total reflectance Fourier transform infrared (ATR-FTIR) spectroscopy was collected on a Thermo Scientific Nicolet 380 FT-IR spectrophotometer. ATR-FTIR instrument was equipped with a Harrick Scientific GATR accessory and a Ge crystal for surface analysis. The MALDI-TOF MS spectrum acquisition for the characterization of RGD-CD molecule was conducted on a Shimadzu AXIMA Performance Instrument (Shimadzu Biotech, Manchester, U.K.) equipped with a 337 nm N_2_-laser. The spectrum was acquired in the positive ion reflection mode. 2,5-Dihydroxybenzoic acid (10 mg/mL) in acetonitrile/water (1:1 v/v) with the cationization agent (sodium chloride–NaCl) was used as a matrix. Static water contact angle measurements were performed using a CAM 101 KSV goniometer. Images were obtained using an integrated digital camera observing the deposited surface of the deionized water (5 μL). Data process and contact angle determination were carried out by using CAM2008 software. Contact angle values were taken from three different locations of each sample, and average data were reported. X-ray photoelectron spectroscopy (XPS) was carried out using a K-Alpha instrument (Thermo Scientific). Fluorescence images were obtained using a Zeiss Observer Z1 fluorescence microscope with an Axiocam MRc5 camera (ZEISS Fluorescence Microscopy, Carl Zeiss Canada Ltd., Canada). The polymer film thickness was determined using atomic force microscopy using a CoreAFM instrument (Nanosurf, Switzerland).

### Synthesis of Ada-Containing DEGMEMA-AdaMA-TMSPMA (P(D_*x*_-A_*y*-_T_*z*_)) Copolymers

For the synthesis of poly(DEGMEMA_75_-AdaMA_20_-TMSPMA_5_) (P(D_75_-A_20_-T_5_)), DEGMEMA (604 mg, 3.21 mmol), TMSPMA (53 mg, 0.215 mmol), and AdaMA (200 mg, 0.854 mmol) were taken in a round-bottom flask with a magnetic bar and purged with nitrogen. Under nitrogen, anhydrous DMF (8 mL) was added, and the monomer solution was degassed with nitrogen. 4-Cyano-4-(thiobenzoylthio)pentanoic acid (9.4 mg, 0.033 mmol) as a chain transfer reagent and AIBN (1.08 mg, 6.6 × 10^–3^ mmol) as initiator was added to the mixture. Subsequently, the reaction flask was sealed and heated at 70 °C for 19 h. After 19 h, the reaction mixture was cooled to room temperature, and DMF was removed under reduced pressure. The crude was redissolved with minimum CH_2_CI_2_, and the polymer was precipitated in cold diethyl ether to remove unreacted monomers. Molecular weight (*M*_n_) and molecular weight distribution (PDI) were determined by size exclusion chromatography (SEC) (*M*_n_ = 20 kDa; PDI (*M*_w_/*M*_n_) = 1.36). Since the proton resonances from the TMS-monomer overlaps with other protons, it is difficult to estimate the amount of TMSPMA incorporation. However, since it is only 5% in the feed, as an approximation, we ignored its contribution. The peak from Ada at 2.01 ppm was used for integration (half peak used for ensuring no overlap) and compared to the OCH_3_ peak at 3.39 ppm, from the DEGMEMA monomer. Using this comparison, a ratio close to the feed ratio was observed, as given in Table 1. A similar experimental procedure was followed for obtaining the polymer for control studies, poly(DEGMEMA_95_-AdaMA_0_-TMSPMA_5_) (P(D_95_-A_0_-T_5_)). Molecular weight (*M*_n_) and molecular weight distribution (PDI) for the control polymer were determined using size exclusion chromatography (SEC) (*M*_n_ = 23 kDa; PDI (*M*_w_/*M*_n_) = 1.40).

**Table 1:** Polymerization Conditions and Characterization Data of AdaMA-DEGMEMA Copolymers

entry	polymer^a^	*F*_theo._^b^	*F*_calcd_^c^	conv. (%)	*M*_n,theo_ (g/mol)	*M*_n,NM*R*_ (g/mol)	*M*_n,SEC_^d^ (g/mol)	*M*_w_*/M*_n_^d^
1	P(D_75_-A_20_-T_5_)	3.75:1	3.17:1	82	21,275	23,581	20,000	1.36
2	P(D_95_-A_0_-T_5_)	1:0	1:0	85	20,301	24,040	23,000	1.40

a [AIBN]_o_/[CTA]_o_: 1/5; [M]_o_: 0.5 M; [M]_o_/[CTA]: 127.

b F_theo._: [DEGMEMA]:[AdaMA].

c F_NMR._: [DEGMEMA]:[AdaMA], using ^1^H NMR.

d Using SEC eluted with DMAC with PMMA standards.

### Cleaning Procedure for Silicon Surfaces

Silicon surfaces (1 × 1 cm^2^) were cleaned using water (5 mL), acetone (5 mL), and ethanol (5 mL), respectively. They were sonicated in these solvents for 15 min and then dried under a stream of nitrogen. Finally, silicon surfaces were cleaned using the Novascan PSD series UV/Digital ozone system for 15 min.

### Fabrication of Polymeric Coating on Silicon Surfaces

Two polymer solutions of P(D_75_-A_20_-T_5_) and P(D_95_-A_0_-T_5_) (50 μL, 20 mg/mL in anhydrous DMF) were spread over each silicon surface. They were left open to air for 24 h at room temperature to let the solvent evaporate. Surfaces were placed into the vacuum oven at 60 °C for 1 h. Finally, surfaces were washed and sonicated in DMF and THF for 2 min to remove the nonadhered polymer from the surface, and then, wafers were dried under a stream of nitrogen.

### Synthesis of βCD-Lissamine Rhodamine-B Conjugate

βCD-NH_2_ (40 mg, 0.034 mmol) and lissamine rhodamine B sulfonyl chloride (30 mg, 0.05 mmol) were dissolved in 0.8 mL of anhydrous DMF. After adding *N*,*N*-diisopropylethyl amine (DIPEA) (24 μL, 0.136 mmol) in 0.2 mL of anhydrous DMF, the mixture was stirred for 2 days at room temperature. At the end of 2 days, the mixture was added to acetone (50 mL), and the precipitated solid residue was centrifuged and washed with an excess amount of acetone. The product was obtained as a purple solid. ^1^H-NMR (D_2_O, δ, ppm) 8.51 (s, 1H), 8.17 (d, *J* = 9.5, 1H), 7.54 (d, *J* = 7.8, 1H), 7.05–6.88 (m, 5H), 6.77 (s, 1H), 5.05 (s, 7H), 3.91–3.44 (m, 50H), 1.34–1.24 (m, 12H).

### Micro-Contact Printing (μCP) of βCD-Dye

A solution of βCD-grafted lissamine rhodamine B dye in water (1 mg/mL) was used to wet a 1 × 1 cm^2^ PDMS stamp. The stamp was left to dry for 60 min, followed by further drying under a gentle stream of N2. The stamp was placed on the polymer-coated S(D_75_-A_20_-T_5_) surfaces for 60 min. After printing, surfaces were washed with cold water to remove nonconjugated materials. The S(D_95_-A_0_-T_5_) surface was used as a control because it did not include the adamantane group to make a complex with the βCD ligand.

### Surface Functionalization with βCD Grafted-Biotin and Immobilization of Qdot-Streptavidin via Micro-Contact Printing

βCD-grafted-biotin solution (50 μL, 2 mg/mL in water) was spread over Ada-containing S(D_75_-A_20_-T_5_) surfaces and control S(D_95_-A_0_-T_5_) surfaces and incubated for 24 h. After incubation, surfaces were washed with cold water to remove unconjugated βCD grafted-biotin. For the immobilization of streptavidin, 10 μL of Qdots-streptavidin was diluted with 90 μL of water, and 10 μL from this solution was spread over a 1 × 1 cm^2^ PDMS stamp. The stamp was left to dry for 60 min, followed by further drying under a gentle stream of N2. The stamp was placed onto the polymer-coated surfaces for 60 min. After printing, surfaces were washed with cold water to remove nonconjugated materials.

### Synthesis of βCD-Mannose

Mono-mannose substituted βCD was synthesized by adapting a literature procedure.^61^ Briefly, the mixture of mannose-alkyne and 6-monodeoxy-6-monoazido-βCD (βCD-N_3_) in DMF was mixed with CuSO_4_.5H_2_O and sodium ascorbate in H_2_O. The mixture was stirred at 60 °C overnight. Afterward, DMF was removed, and a concentrated mixture was added to acetone (50 mL). The formed residue was washed with acetone (50 mL × 3 times). The solid product was redissolved in water (5 mL), and the copper-removing resin (CupriSorb) was added. The mixture was stirred until the color mixture transformed from blue to colorless. Finally, the mixture was filtered to remove resin and lyophilized after freezing (pale yellow solid, 90% yield) ^1^H-NMR (D_2_O, δ, ppm) 8.46 (s, 1H), 5.08 (br s, 8H), 4.02–3.81 (m, 32H), 3.67–3.57 (m, 16H).

### Surface Functionalization with βCD-Mannose and Immobilization of FITC-ConA and Rhodamine-PNA Conjugates

βCD-mannose solution (50 μL, 2 mg/mL in water) was spread over the Ada-containing polymer-coated S(D_75_-A_20_-T_5_) and control S(D_95_-A_0_-T_5_) surface, and surfaces were incubated for 24 h. After incubation, surfaces were washed with cold water to remove unconjugated βCD-Man. FITC-ConA and rhodamine-PNA conjugates were used to evaluate lectin-sugar interaction. FITC-ConA (10 μL, 0.1 mg/mL in 20 mM HEPES buffer) was spread over PDMS stamps (1 × 1 cm^2^). The stamps were left to dry for 60 min, followed by further drying under a gentle stream of N_2_. The stamps were placed onto the S(D_75_-A_20_-T_5_) and S(D_95_-A_0_-T_5_) polymer-coated surfaces for 60 min. After printing, surfaces were washed with cold water to remove nonconjugated materials. Likewise, RhB-PNA solution (0.15 μg/mL in 20 mM HEPES buffer) was spread over a PDMS stamp (1 × 1 cm^2^). After drying, the stamp was placed on the S(D_75_-A_20_-T_5_) surface for 60 min. After printing, surfaces were washed with cold water to remove nonconjugated materials.

### Synthesis of cRGD-βCD

βCD-SH was synthesized according to the literature.^62^ βCD-SH (1.6 mg, 1.39 × 10^–3^ mmol) and maleimide-terminated cyclic RGD peptide (cRGD-Mal) (1 mg, 1.32 × 10^–3^ mmol) were dissolved in anhydrous DMF (0.2 mL), and Et_3_N (0.2 μL, 0.14 mg, 1.39 × 10^–3^ mmol) was added. The mixture was stirred at room temperature overnight. After that, the reaction mixture was concentrated and precipitated in cold Et_2_O (white solid, 80% yield). ^1^H-NMR (D_2_O, δ, ppm): 7.47–7.25 (m, 5H), 5.07 (br s, 7H), 4.00–3.74 (m, 28H), 3.69–3.53 (m, 14H). MALDI-TOF (*m*/*z*) calcd for C_76_H_116_N_10_O_44_S [M]^+^ 1904.69, found [M + Na + H_2_O] 1945.35 g/mol.

### Surface Functionalization with cRGD-βCD

The βCD-cRGD solution (50 μL, 2 mg/mL in water) was spread over Ada-containing S(D_75_-A_20_-T_5_) and control S(D_95_-A_0_-T_5_) surfaces, and surfaces were incubated for 24 h. Finally, surfaces were washed with cold water to remove unconjugated cRGD-βCD.

### Cell Adhesion on Peptide-Functionalized Surfaces

L929 mouse fibroblast cells were grown in a 5% CO_2_-containing atmosphere at 37 °C and cultured in Dulbecco’s Modified Eagle’s Medium (DMEM) media supplemented with 10% fetal bovine serum (FBS) (PAN Biotech). Cells (15,000 cells/30 μL) were seeded onto polymer-coated silicon surfaces (1 × 1 cm^2^) and incubated at 37 °C in a 5% CO_2_-containing atmosphere for 2 h. Subsequently, DMEM (1 mL) was added to each well containing the polymer-coated silicon wafers. After 24 and 48 h incubation, cell media was removed, silicon surfaces were washed with 1 × PBS (0.5 mL, two times), and cells were fixed with 3.7% formaldehyde solution in PBS for 10 min at room temperature. After fixation, cells were washed with 1 × PBS (0.5 mL, two times). For staining filamentous actins (F-actins), cells were incubated with 0.1% Triton X-100 in PBS for 5 min at 37 °C. After washing with 1 × PBS (0.5 mL, two times), cells were incubated with Alexa Fluor 488 phalloidin solution (5 units/mL) in PBS, including 1% bovine serum albumin (BSA) for 15 min at 37 °C. After washing with PBS, cell nuclei were stained with DAPI for 10 min at 37 °C. After the staining steps, the stained cells were washed with 1 × PBS. The resulting images of stained cells on polymer-coated silicon surfaces were taken using a fluorescence microscope, and processing was carried out using a Zeiss Zen Blue lite software (Carl Zeiss, Jena, Germany).

## Results and Discussion

### Synthesis of Copolymers Containing Adamantane (Ada) Side Chains

**Figure 1:**
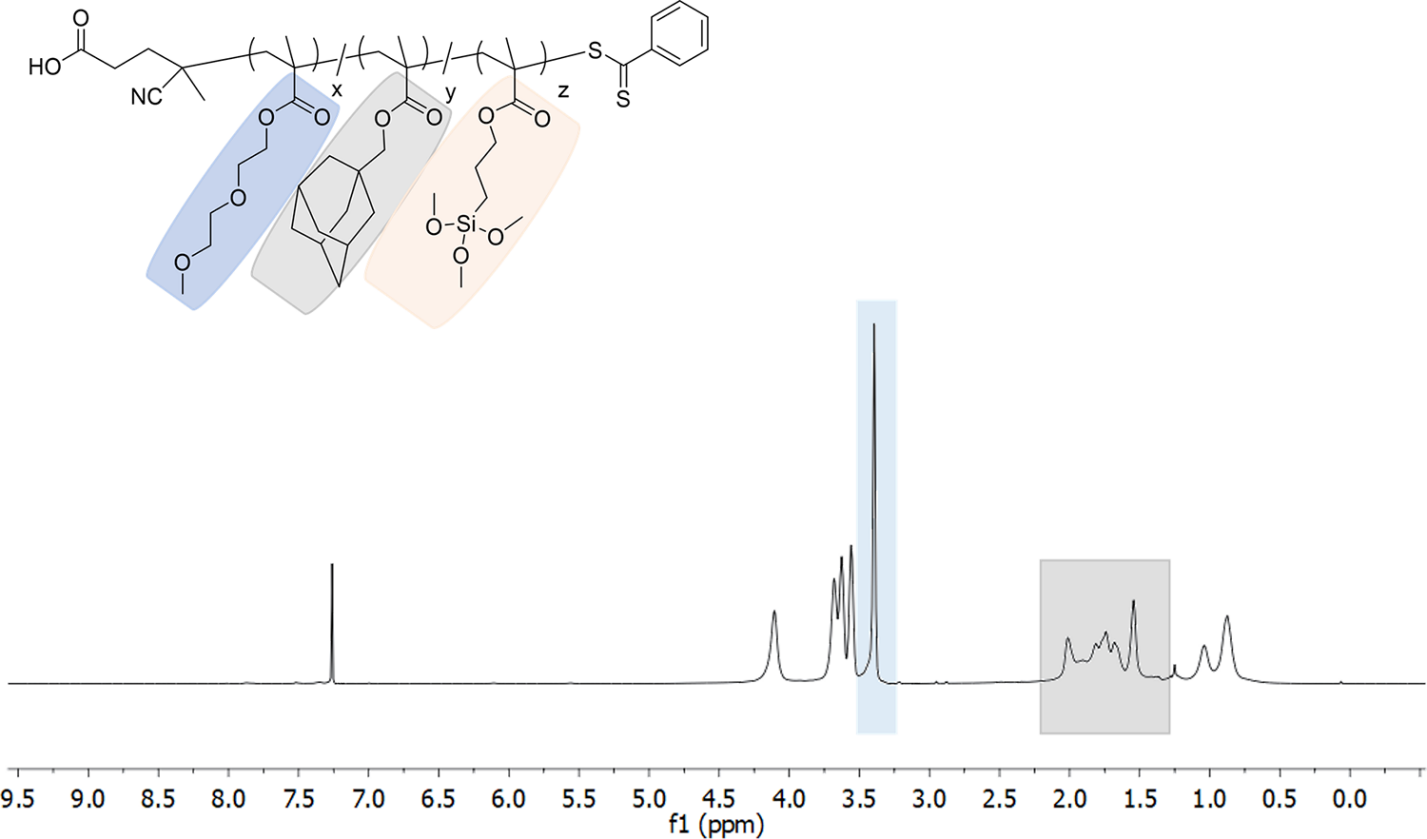
^1^H NMR spectrum of P(D_75_-A_20_-T_5_) copolymer (CDCl_3_).

**Scheme 2:**
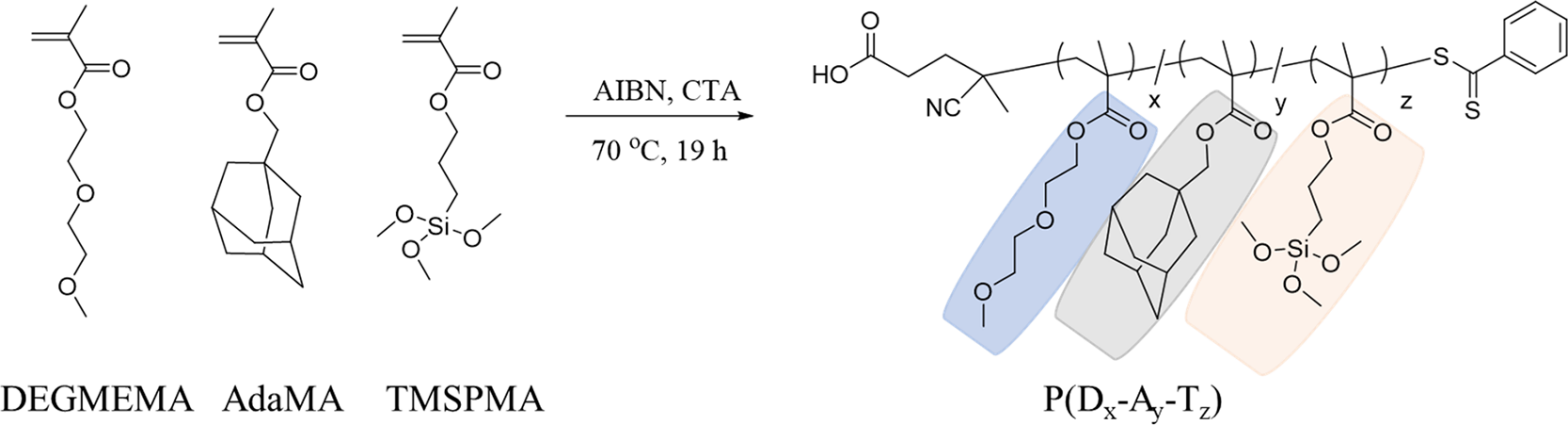
Synthesis of Surface-Anchorable Ada-Containing Copolymers

For the synthesis of copolymers bearing Ada units, adamantane methacrylate (AdaMA) was synthesized and copolymerized with a hydrophilic di(ethylene glycol)-based methacrylic monomer (DEGMEMA), and surface active trimethoxysilyl-based methacrylic monomer (TMSPMA) using RAFT polymerization (Scheme 2). Copolymers containing the AdaMA, TMSPMA, and DEGMEMA monomers ((P(D_*x*_-A_*y*_-T_*z*_)) where *x*, *y*, and *z* subscript numbers show their mol percentage in the feed) were synthesized. Two different copolymers, P(D_95_-A_0_-T_5_) and P(D_75_-A_20_-T_5_), with 0 and 20% Ada groups, respectively, were synthesized (Table 1). The TMSPMA monomer ratio was kept at 5%, sufficient for surface attachment.^63^ Copolymer P(D_95_-A_0_-T_5_), devoid of any Ada groups, was synthesized for control experiments to deduce the role of the Ada group in surface functionalization through complexation with CD. After the polymerization, copolymers were purified by precipitating in cold diethyl ether and were characterized by SEC and ^1^H NMR spectroscopy. Obtained copolymers showed high monomer conversions, and SEC analysis demonstrated reasonable control on molecular weight during polymerization with relatively narrow molecular weight distribution. Successful incorporation of monomers in the obtained copolymers was deduced using ^1^H NMR spectroscopy (Figures 1 and S2). For copolymer P(D_75_-A_20_-T_5_), proton resonance from the methoxy groups (−OCH_3_) of DEGMEMA was observed at 3.39 ppm, along with the proton resonances of adamantane hydrogens between 2.01 and 1.54 ppm. Interestingly, the proton resonances from the silyl-containing monomers were not visible, most likely due to peak overlaps. Notably, copolymers synthesized without any silyl-based monomer to the feed did not show any surface adhesion in subsequent surface modification studies.

### Fabrication and Characterization of Polymer Coatings on Silicon Surfaces

**Figure 2:**
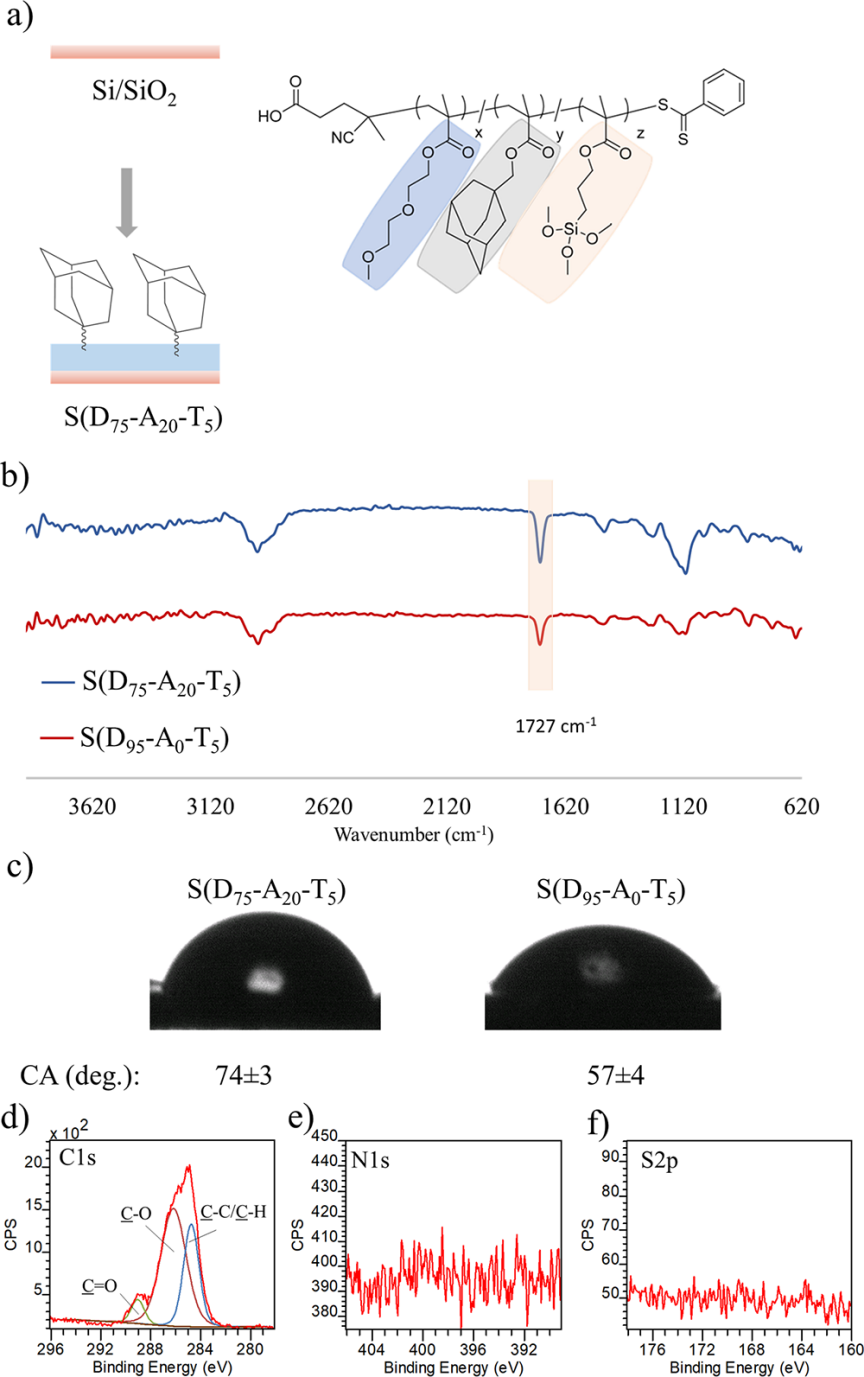
(a) Schematic illustration of coating of polymers onto a silicon surface, (b) ATR-FTIR spectrum of polymer-coated surfaces, (c) water contact angle of polymer-coated surfaces and high-resolution XPS spectra (d) C1s, (e) N1s, and (f) S2p of the S(D_75_-A_20_-T_5_) surface.

Cleaned Si/SiO_2_ surfaces were used to fabricate polymer coating on surfaces. P(D_75_-A_20_-T_5_) and P(D_95_-A_0_-T_5_) polymer solutions were prepared in anhydrous DMF (20 mg/mL). After spreading polymer solutions over Si/SiO_2_ surfaces, incubating, and heating at 60 °C for 60 min, surfaces were rinsed with copious amounts of organic solvents to remove unbound polymers. Polymer-coated surfaces obtained using P(D_75_-A_20_-T_5_) and P(D_95_-A_0_-T_5_) are referred to as S(D_75_-A_20_-T_5_) and S(D_95_-A_0_-T_5_) in subsequent sections, and “S” stands for “surface”. The coated surfaces were analyzed using FT-IR spectroscopy. The presence of the characteristic carbonyl bands around 1727 cm^–1^ suggested the successful attachment of polymers onto the Si/SiO_2_ surfaces (Figure 2b). In addition to the FT-IR analysis, contact angle measurements recorded for S(D_75_-A_20_-T_5_) and S(D_95_-A_0_-T_5_) also suggested the same. While the water contact angle of S(D_95_-A_0_-T_5_) was found as 57 ± 4, that of S(D_75_-A_20_-T_5_) was determined as 74 ± 4, presumably due to the hydrophobic nature of the adamantane groups (Figure 2c), as well as decrease in the content of the ethylene-glycol based monomer. Further characterization of S(D_75_-A_20_-T_5_) was undertaken with XPS analysis. The survey scan of S(D_75_-A_20_-T_5_) comprises C1s and O1s signals 285.0 and 532.5 eV. The high-resolution C1s spectrum could be deconvoluted to three Gaussians with the expected relative areas for three carbon atoms at 285.0 eV (C–C/C–H), 286.4 eV (C–O–C/C–N/C–S), and 289.3 eV (C=O) (Figure 2d). Importantly, no significant N and S atoms peaks were visible, presumably due to the very low concentration since they are only present as end groups in the copolymer synthesized using RAFT polymerization (Figure 2e,f). The lack of peaks from these atoms in the native polymer coating will be critical to our analysis after their subsequent functionalization using host–guest interactions. Additionally, very small peaks belonging to Si were visible (Figure S3), but this is expected since the polymer contains silyl groups. In addition, the thickness and surface roughness (*R*_a_) of S(D_75_-A_20_-T_5_) surface were found as 71 ± 16 nm and 4.2 ± 1.2 nm using AFM analysis (*R*_a_ = 0.64 ± 0.14 nm for uncoated silicon surface). After characterization, the stability of polymer coatings in aqueous media was investigated. S(D_75_-A_20_-T_5_) and S(D_95_-A_0_-T_5_) surfaces were incubated in PBS solution (pH = 7.4) at 37 °C for 24 h. Dried surfaces were characterized using FT-IR spectroscopy. No significant change in the carbonyl peaks after incubation with PBS suggested that polymer coatings were stable in an aqueous environment (Figure S4).

#### Functionalization of Ada-Containing Coatings Using Host–Guest Interactions

##### Immobilization of βCD-Lissamine Rhodamine B Dye

**Figure 3:**
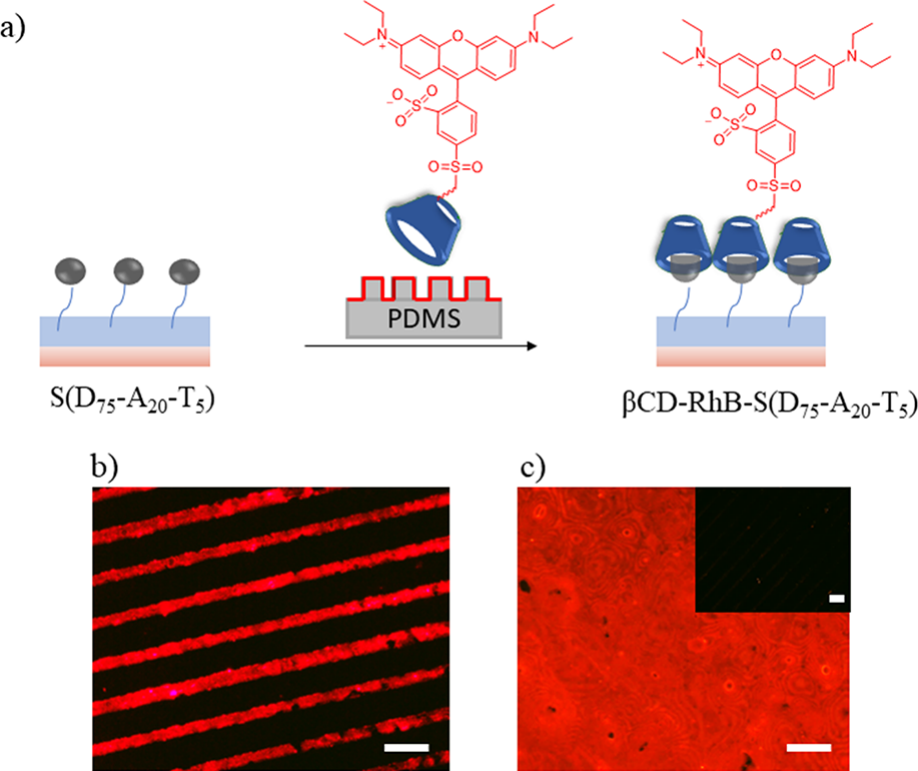
(a) Schematic illustration of polymer-coated surface modification via μCP using βCD-grafted lissamine rhodamine B dye; fluorescence microscopy images of dye-modified (b) patterned surfaces, (c) nonpatterned surfaces, and inset S(D_95_-A_0_-T_5_). The scale bar is 100 μm.

Initial functionalization of S(D_75_-A_20_-T_5_) and S(D_95_-A_0_-T_5_) was undertaken with βCD-lissamine rhodamine B via the μCP method using a PDMS stamp (Figure 3a). The S(D_75_-A_20_-T_5_) surface was brought in conformal contact with a patterned PDMS stamp inked with the dye. After printing, the surface was washed with cold water to remove any unbound dye. For the control experiment, the S(D_95_-A_0_-T_5_) surface was used and treated with βCD-lissamine rhodamine B dye in the same manner. After printing, surfaces were visualized under a fluorescence microscope. While S(D_75_-A_20_-T_5_) surface revealed red fluorescence (Figure 3b,c), S(D_95_-A_0_-T_5_) did not show any fluorescent pattern, presumably due to the lack of host–guest interaction on the surface devoid of any adamantane groups (Figure 3c, inset). These results suggest that obtained polymer-coated interfaces are amenable to facile functionalization using specific noncovalent interactions in an aqueous environment. Furthermore, surface S(D_75_-A_20_-T_5_) modified with βCD-grafted lissamine rhodamine B dye was immersed in PBS buffer (pH 7.4) and incubated at 37 °C for 48 h and analyzed using fluorescence microscopy at predetermined time intervals. The fluorescence intensity of the βCD-grafted lissamine rhodamine B modified S(D_75_-A_20_-T_5_) surface showed no significant change when incubated at 37 °C for 48 h (Figure S5). Observations suggest significant stability of thus modified polymer surface βCD/Ada-based host–guest interactions.

##### Biomolecular Immobilization on Polymer Coatings

**Figure 4:**
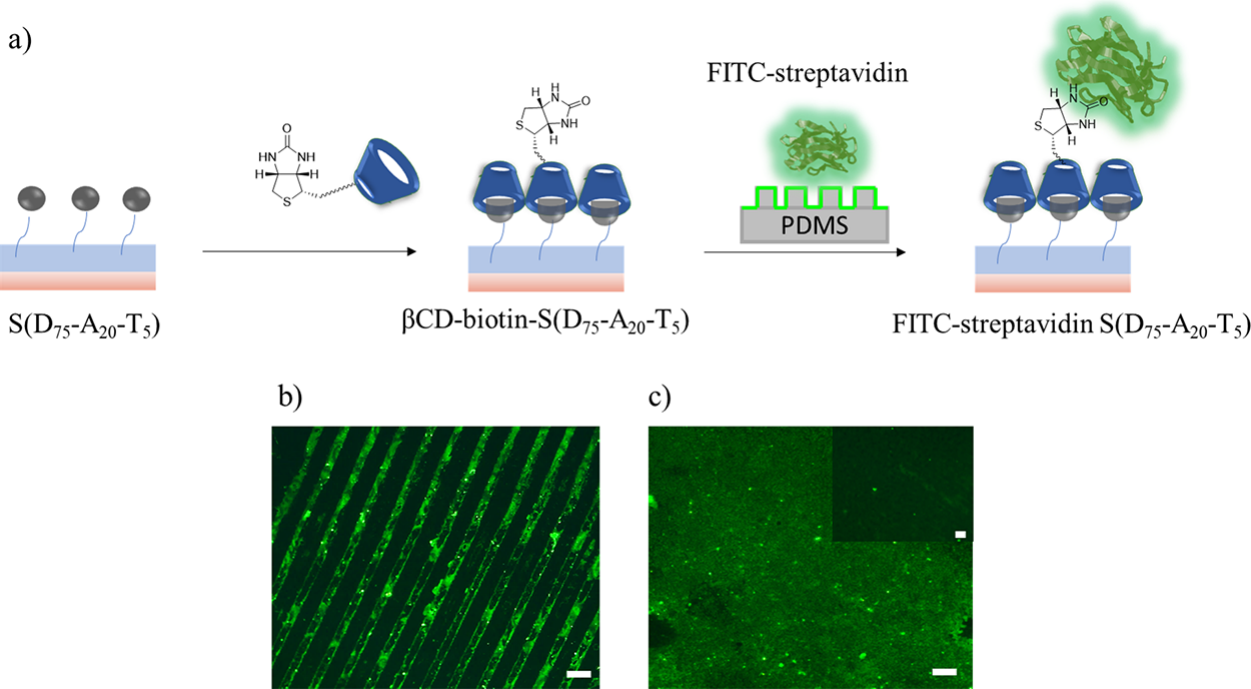
(a) Schematic illustration of polymer-coated surface modification with βCD-grafted biotin and immobilization of FITC-streptavidin using μCP, fluorescence microscopy images of CdSe Qdot streptavidin-modified surfaces (b) patterned S(D_75_-A_20_-T_5_) and (c) nonpatterned S(D_75_-A_20_-T_5_). The scale bar is 50 μm.

To probe the efficiency of adamantane-containing polymer film for attachment of bioactive ligands at the surface, conjugation of CD-containing biotin ligands was examined. Polymeric films functionalized with bioactive ligands can be used to detect the presence of a target analyte like specific proteins or can be utilized to immobilize biomolecules for specific applications. FITC-streptavidin conjugates were selected as a model protein to investigate biomolecular immobilization using strong noncovalent biotin–streptavidin interaction (*K*_d_ = 1 × 10^–14^ M).^64^ To this end, a βCD-biotin conjugated S(D_75_-A_20_-T_5_) surface was modified with FITC-streptavidin with and without a pattern (Figure 4a). Surface S(D_95_-A_0_-T_5_), devoid of any adamantyl group, was used for control experiments. In addition to the S(D_95_-A_0_-T_5_) surface, the S(D_75_-A_20_-T_5_) surface, which was not modified with βCD-biotin, was also utilized for the control experiment. After treatment of the biotinylated surface with FITC-streptavidin, surfaces were washed with PBS to remove any unconjugated material. Upon visualization under a fluorescence microscope, green fluorescence was obtained for both patterned and nonpatterned biotinylated surfaces (Figure 4b,c). As expected, control surfaces did not exhibit any significant green fluorescence (Figure 4c, inset).

**Figure 5:**
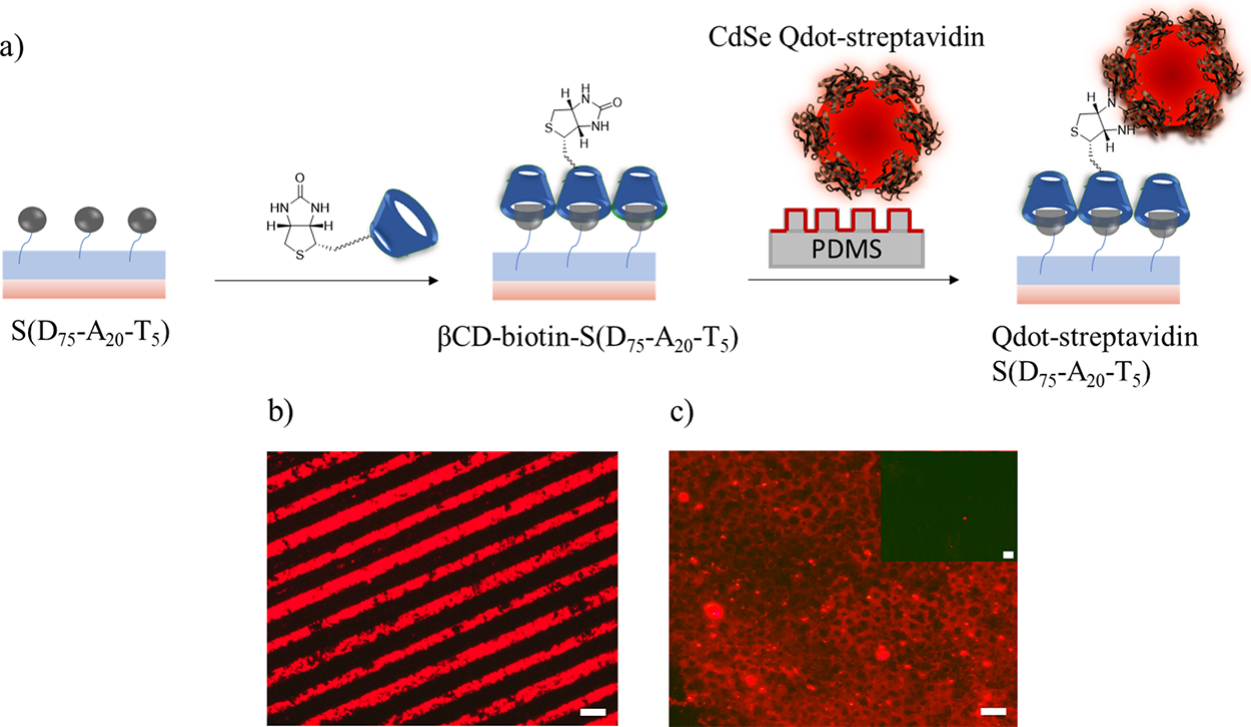
(a) Schematic illustration of polymer-coated surface modification with βCD-grafted biotin and immobilization of CdSe Qdot-streptavidin using μCP; (b) fluorescence microscopy image of the CdSe Qdot-streptavidin-modified patterned S(D_75_-A_20_-T_5_) surface; (c) fluorescence microscopy image of the CdSe Qdot-streptavidin-modified nonpatterned S(D_75_-A_20_-T_5_) surface. The scale bar is 50 μm.

After the conjugation of FITC-streptavidin onto biotin-immobilized S(D_75_-A_20_-T_5_), further modification was demonstrated using the CdSe Qdot-streptavidin conjugate. This was undertaken to demonstrate that such functionalizable interfaces can be also modified with comparatively large inorganic nanoparticles. In the first step, βCD-grafted biotin (βCD-biotin) was immobilized onto S(D_75_-A_20_-T_5_) by host–guest interactions. After immobilization, the surface was washed with cold water to remove unbound βCD-biotin. Also, the control surface S(D_95_-A_0–_T_5_) was treated with βCD-biotin similarly. After conjugation of βCD-biotin, the Qdot-streptavidin conjugate was immobilized onto a biotinylated and control surface with μCP (Figure 5a). After printing, surfaces were washed with cold water to remove nonconjugated materials. Upon visualization under a fluorescence microscope, a bright red fluorescence was observed on both patterned and nonpatterned biotinylated surface S(D_75_-A_20_-T_5_) (Figure 5b,c). As expected, the control surface S(D_95_-A_0_-T_5_) revealed no fluorescence since no binding occurred due to the lack of adamantane groups (Figure 5c, inset).

**Figure 6:**
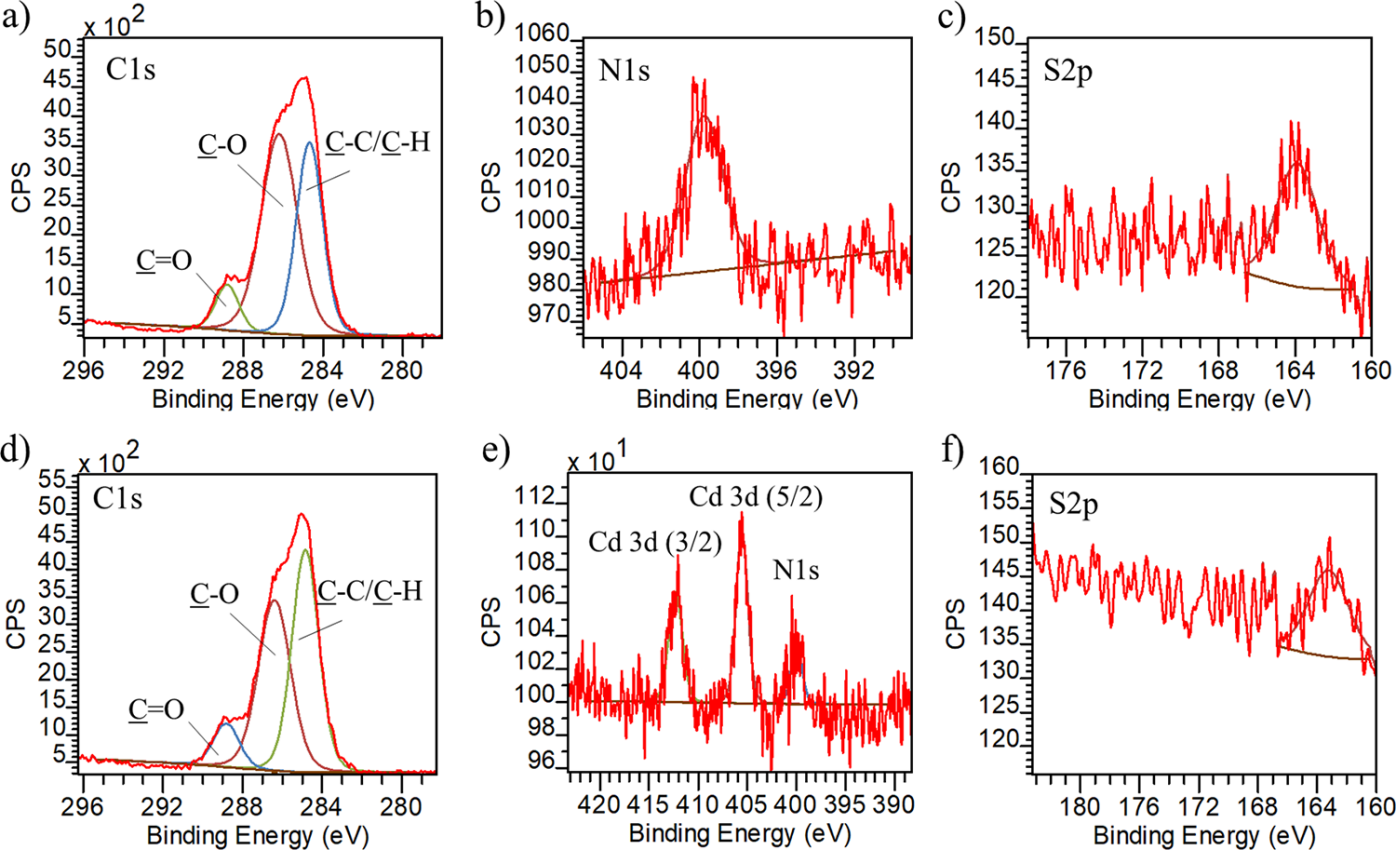
High-resolution XPS spectra (a) C1s, (b) N1s, and (c) S2p of βCD-biotin conjugated S(D_75_-A_20_-T_5_) and high-resolution XPS spectra (d) C1s, (e) Cd3d and N1s, and (f) S2p of βCD-biotin conjugated S(D_75_-A_20_-T_5_) after immobilization of CdSe-Qdot conjugated streptavidin.

Biotin-conjugated surface S(D_75_-A_20_-T_5_) was also characterized by XPS. The survey scan of biotin-conjugated S(D_75_-A_20_-T_5_) comprises C1s and O1s signals 285.0 and 532 eV. The high-resolution C1s spectrum could be deconvoluted to three Gaussians with the expected relative areas for three carbon atoms at 285.0 eV (C–C/C–H), 286.2 eV (C–O–C/C–N/C–S), and 288.8 eV (C=O) (Figure 6a). Figure 6b shows the high-resolution XPS N1s spectra of the biotin-conjugated S(D_75_-A_20_-T_5_) surface, where the characteristic N atom peak was visible at 400.0 eV. In addition, the S2p peak coming from biotin was also observed at 164.2 eV (Figure 6c). N1s and S2p signals, which were similar to previously reported literature data,^65^ suggest successful immobilization of βCD-biotin onto the Ada-containing S(D_75_-A_20_-T_5_) via host–guest interactions. In addition to fluorescence microscopy investigations, CdSe Qdot-streptavidin conjugated biotin-S(D_75_-A_20_-T_5_) was also characterized by XPS. The characteristic N1s (Figure 6e) and S2p (Figure 6f) peaks from biotin were observed at 400 and 164.2 eV, respectively. In addition to these peaks, the characteristic Cd signals were visible at 412.2 eV (Cd 3d 3/2) and 405.3 eV (Cd 3d 5/2), and Cd signals were similar to those observed in the literature (Figure 6e).^66^

##### Lectin Binding to Mannose-Containing Polymer Coatings

**Figure 7:**
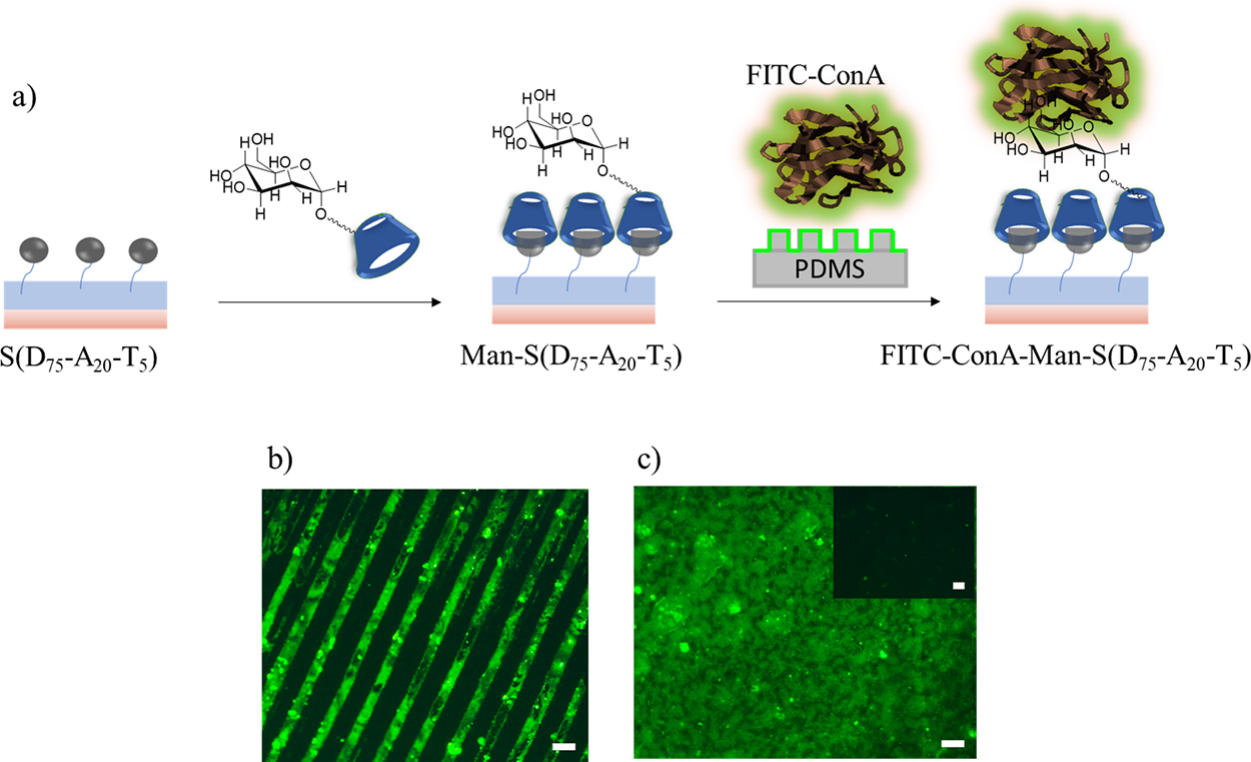
(a) Schematic illustration of polymer-coated surface modification with βCD-grafted mannose and immobilization of FITC-ConA using μCP; (b) fluorescence microscopy image of FITC-ConA-modified patterned S(D_75_-A_20_-T_5_) surface; (c) fluorescence microscopy image of FITC-ConA-modified nonpatterned S(D_75_-A_20_-T_5_) surface. The scale bar is 50 μm.

**Figure 8:**
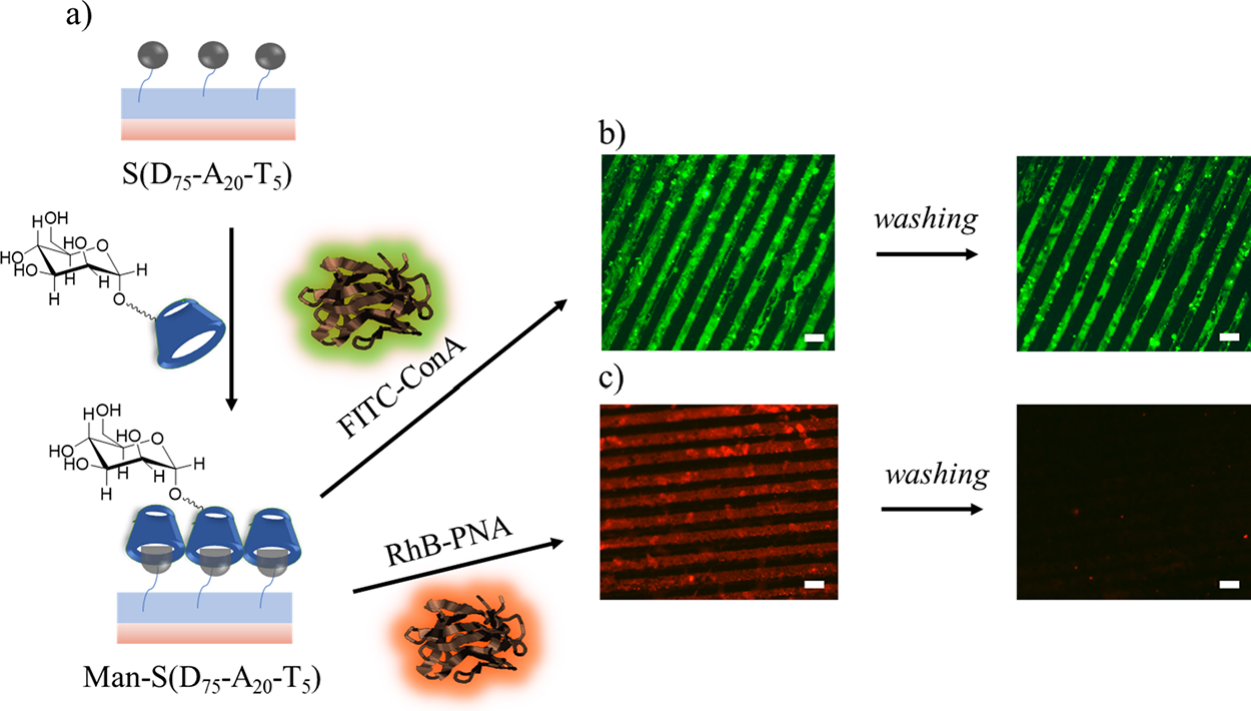
(a) Schematic illustration of polymer-coated surface modification with βCD-grafted mannose and immobilization of FITC-ConA using μCP; fluorescence microscopy images of (b) FITC-ConA-modified and (c) Rhodamine-PNA-modified S(D_75_-A_20_-T_5_) surfaces before and after washing steps. The scale bar is 50 μm.

A surface decorated with mannose was obtained using immobilization of a mannose-containing CD, followed by investigating its ability to bind selectively to a dye-labeled lectin, namely, fluorescein-labeled concanavalin A (FITC-ConA). For this purpose, Ada-containing surface S(D_75_-A_20_-T_5_) and control surface S(D_95_-A_0_-T_5_) were treated with βCD-bearing mannose. Unbound βCD-bearing mannose was removed from the surface by rinsing the surfaces with water. βCD-Man-S(D_75_-A_20_-T_5_) and control S(D_95_-A_0_-T_5_) surfaces were subsequently evaluated for Con A binding (Figure 7a). βCD-Man-S(D_75_-A_20_-T_5_) and control S(D_95_-A_0_-T_5_) surfaces brought in conformal contact with a microstructured PDMS stamp primed with a solution of FITC-ConA. After washing with HEPES (20 mM) solution, to remove any physically adhered FITC-ConA, the silicon surface was visualized under a fluorescence microscope. As expected, while the βCD-Man-S(D_75_-A_20_-T_5_) surface displayed green fluorescence with clear patterns (Figure 7b), the control S(D_95_-A_0_-T_5_) surface did not show any fluorescence (Figure 7c, inset). After the demonstration of FITC-ConA lectin binding, the specificity of this immobilization was investigated using rhodamine B-peanut agglutinin (RhB-PNA) lectin. βCD-Man-S(D_75_-A_20_-T_5_) and control S(D_95_-A_0_-T_5_) surfaces were treated with RhB-PNA using the μCP method (Figure 8). After printing, the surface was visualized using a fluorescence microscope. As seen in Figure 8c, a uniform pattern with red fluorescence was observed before a rinsing step. Subsequently, upon rinsing the surface with cold water, the red fluorescent pattern disappeared as expected since the lectin PNA does not possess any binding affinity toward mannose.

**Figure 9:**
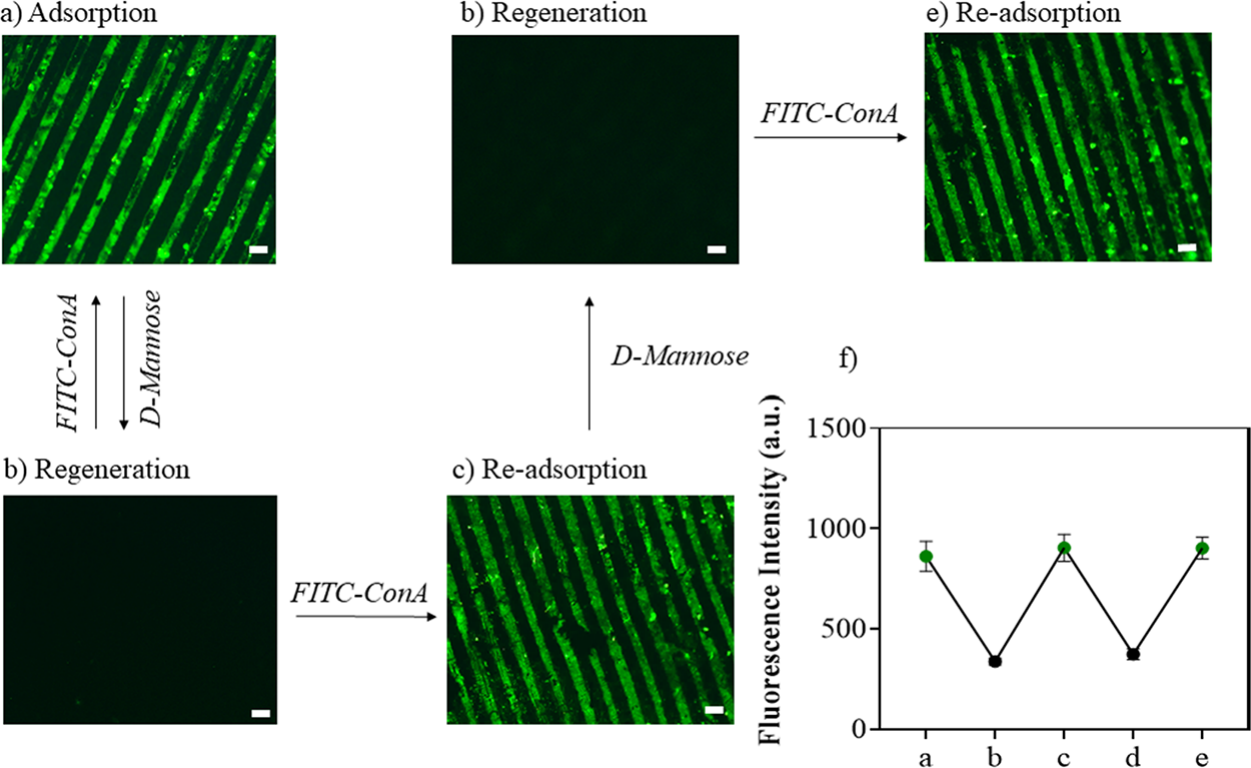
Regeneration and reuse of the βCD-Man-S(D_75_-A_20_-T_5_) surface. (a) Adsorption of FITC-ConA on the βCD-Man-S(D_75_-A_20_-T_5_) surface; (b) regeneration by releasing of ConA in the presence of d-mannose; (c) re-adsorption of FITC-ConA on βCD-Man-S(D_75_-A_20_-T_5_); (d) surface regeneration by releasing of ConA in the presence of d-mannose; (e) re-adsorption of FITC-ConA; and (f) cycles of reuse of βCD-Man-S(D_75_-A_20_-T_5_) were measured by analyzing the mean fluorescence intensity of the images during the cycles.

After demonstrating the successful specific binding of lectin ConA onto the mannose-decorated surface, we investigated if the bound ConA could be released from the surface. Interactions between the βCD-Man-S(D_75_-A_20_-T_5_) surface and conjugated ConA should decrease in the presence of a high concentration of a competitive ligand such as free sugar, and reduced interaction should lead to the release of ConA from the surface. To this end, FITC-ConA printed βCD-Man-S(D_75_-A_20_-T_5_) surfaces were incubated with d-(+)-mannose solution to enable the release of ConA. Upon visualization under a fluorescence microscope, no green fluorescence on the surface was observed after treatment with a d-(+)-mannose solution (Figure 9b,d), suggesting the release of FITC-ConA from the surface. Subsequently, regeneration/reutilization of the βCD-Man-S(D_75_-A_20_-T_5_) surface was also investigated, and it was observed that FITC-ConA could be immobilized onto regenerated βCD-Man-S(D_75_-A_20_-T_5_) surfaces. As seen in Figure 9c,e, the regenerated βCD-Man-S(D_75_-A_20_-T_5_) surface could immobilize FITC-ConA in subsequent cycles with almost similar efficiency (Figure 9f).

##### Cell Attachment on cRGD-Conjugated-βCD-Coated Surfaces

Finally, we wanted to investigate if polymer-coated surfaces could be rendered amenable for cell attachment through noncovalent decoration with cell-adhesive peptides. To this end, cRGD-conjugated βCD was synthesized using maleimide-terminated cRGD and βCD-SH in the presence of triethylamine via the thiol-maleimide Michael addition reaction (Figure S9). S(D_75_-A_20_-T_5_) surfaces were treated with cRGD-conjugated βCD to obtain an interface displaying the cRGD peptide. After incubation of the surface with peptide-containing βCD solution, unbound βCD-derivatives were rinsed from the surface with water. To confirm that the peptide was immobilized onto the surface through host–guest interactions, the peptide-decorated surface S(D_75_-A_20_-T_5_) was characterized by XPS. The survey scan cRGD peptide conjugated S(D_75_-A_20_-T_5_) comprises C1s and O1s signals at 285.0 and 533 eV, respectively (Figure S6). The high-resolution C1s spectrum could be deconvoluted to three Gaussians with the expected relative areas for three carbon atoms at 285.0 eV (C–C/C–H), 286.0 eV (C–O–C/C–N), and 289.1 eV (C=O) (Figure S4c). Notably, the characteristic N1s peak from the cRGD peptide was observed at 400 eV (Figure S6d), confirming the peptide’s successful attachment onto the surface.

**Figure 10:**
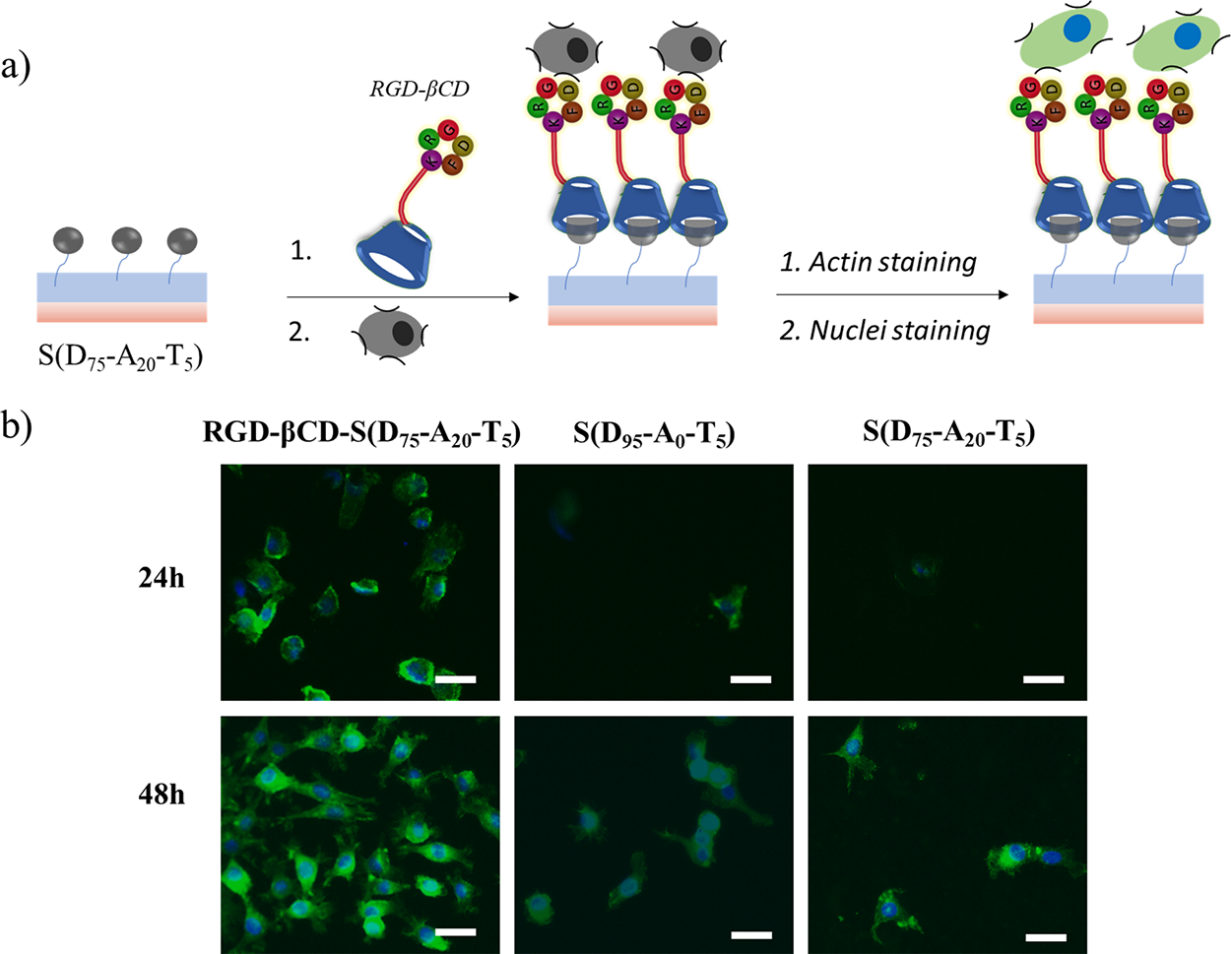
(a) Scheme of conjugation of RGD-βCD and subsequent cellular attachment; (b) merged fluorescence microscopy images of fibroblast cells immobilized on RGD-βCD-S(D_75_-A_20_-T_5_), S(D_95_-A_0_-T_5_), and S(D_75_-A_20_-T_5_) surfaces after actin filament staining with Alexa Fluor 488 and nuclei staining with DAPI. The scale bar is 50 μm.

After the surface immobilization of cRGD-βCD, L929 mouse fibroblast cells were seeded onto the peptide-modified surface (RGD-βCD-S(D_75_-A_20_-T_5_)). S(D_75_-A_20_-T_5_) without RGD attachment and S(D_95_-A_0_-T_5_) surfaces were utilized for the control experiment. After 24 and 48 h incubation, the actin and nuclei of attached cells were stained using an Alexa fluor-phalloidin-488 kit and DAPI, respectively. After the staining processes, images of cells on the polymer-coated surfaces were obtained using a fluorescence microscope. In the case of immobilization of the polymer surface with cRGD-βCD, enhanced cell attachment and proliferation were observed after 24 and 48 h (Figure 10b). As a comparison, the attachment of cells onto S(D_75_-A_20_-T_5_) (not treated with cRGD-βCD) and the control surface S(D_95_-A_0_-T_5_) remained significantly low due to the anti-biofouling character of the PEG-based copolymer coating.

## Conclusions

In summary, the fabrication of a versatile polymeric coating amenable to facile functionalization in a plug-and-play modular manner to immobilize various functional molecules ranging from fluorescent dyes to bioactive ligands is demonstrated. An Ada-CD-based host–guest complexation on a polymeric interface enables a variety of functionalization in a robust and reliable manner. Examples here demonstrate the modification of polymer-coated surfaces for immobilizing fluorescent dyes, specific proteins, and protein-coated inorganic nanocrystals. Protein immobilization/sensing can be undertaken selectively by appropriate choice of the functional molecules at the interface. Through catch-and-release cycles, we demonstrate that obtained interface is robust enough for regeneration and reusability in specific applications. Finally, we show that the inherently anti-biofouling polymeric interface can be tailored for the attachment of cells through modification with cell-adhesive peptides. One can envision that the facile fabrication and versatile and modular nature of the interface engineering demonstrated here will be deemed attractive for fabricating functional devices for various biomedical applications.

## Supporting Information Available

NMR spectra for the copolymer; lissamine RhB βCD, mannose-βCD, and cRGD-βCD; FTIR and XPS spectra; and the result of the stability experiment (PDF)
