# Supplementary material for: Plug-and-Play Biointerfaces: Harnessing Host–Guest Interactions for Fabrication of Functional Polymeric Coatings
Source: Biomacromolecules. 2023 Jul 5;24(8):3568–79. doi: 10.1021/acs.biomac.3c00360 (PMC10428160; doi:10.1021/acs.biomac.3c00360)
Supplement: Supplementary file 1 — bm3c00360_si_001.pdf [file bm3c00360_si_001.pdf]

*Supporting Information*

# Plug-and-Play Biointerfaces: Harnessing Host-Guest Interactions for Fabrication of Functional Polymeric Coatings


*Aysun Degirmenci,[a] Rana Sanyal,[a,b] Amitav Sanyal,[a,b*]*

[a] Department of Chemistry, Bogazici University, Bebek, Istanbul, 34342, Türkiye

[b] Center for Life Sciences and Technologies, Bogazici University, Istanbul, Türkiye

Email: amitav.sanyal@boun.edu.tr

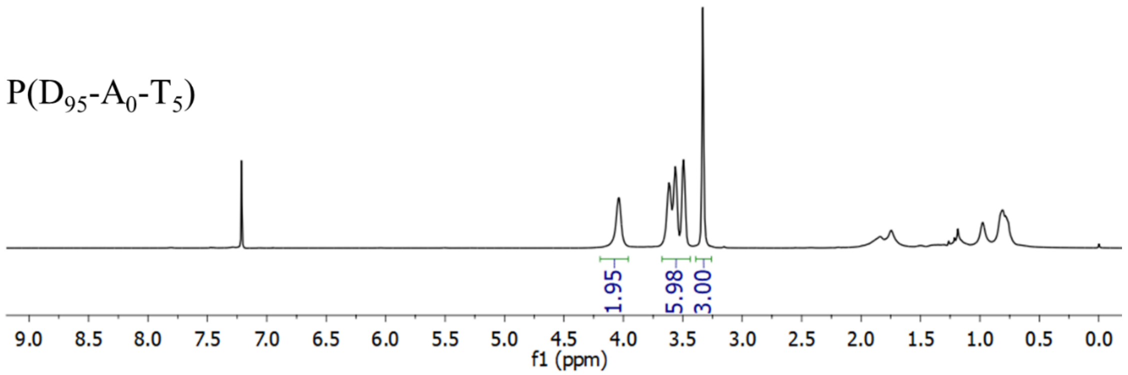


**Figure S1.** $^{1}$H NMR spectrum of P($D_{95}$-$A_0$-$T_5$) copolymer in $CDCl_3$.

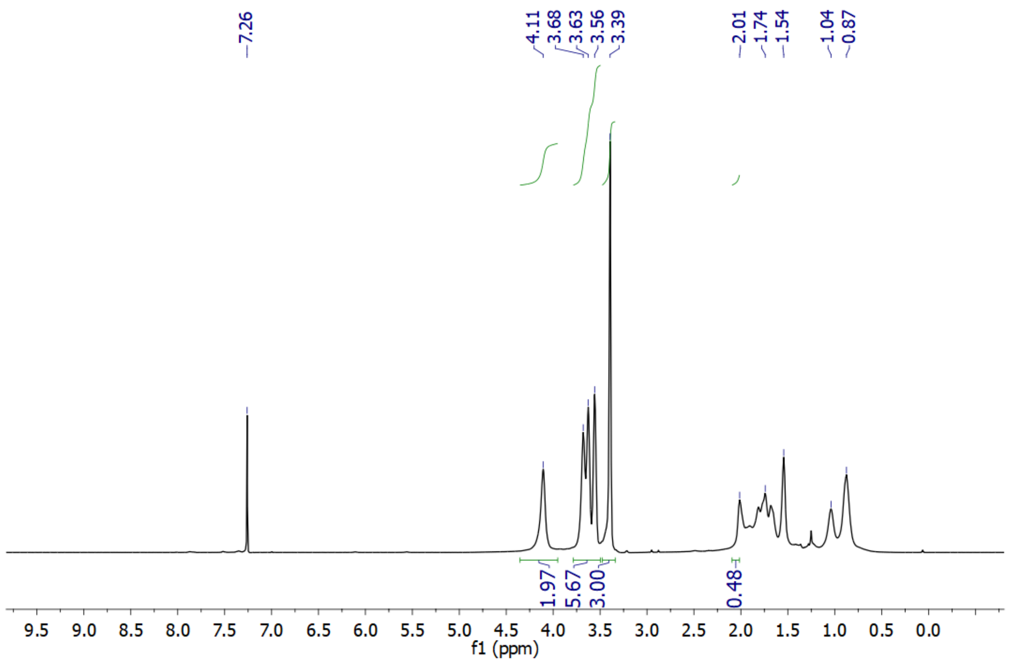


**Figure S2.** $^{1}$H NMR spectrum of P($D_{75}$-$A_{20}$-$T_5$) copolymer in $CDCl_3$.

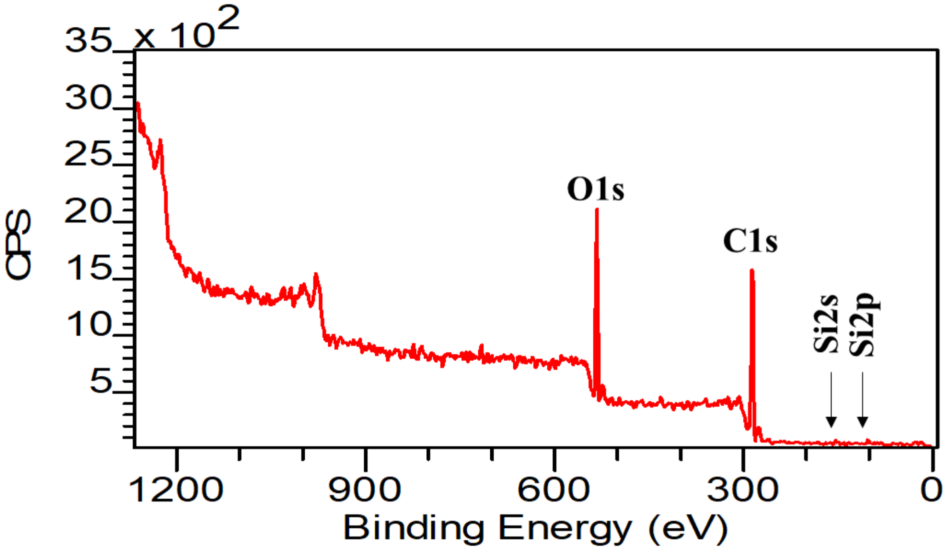


**Figure S3.** Full survey scan XPS spectrum of S(D75-A20-T5) coated surface.

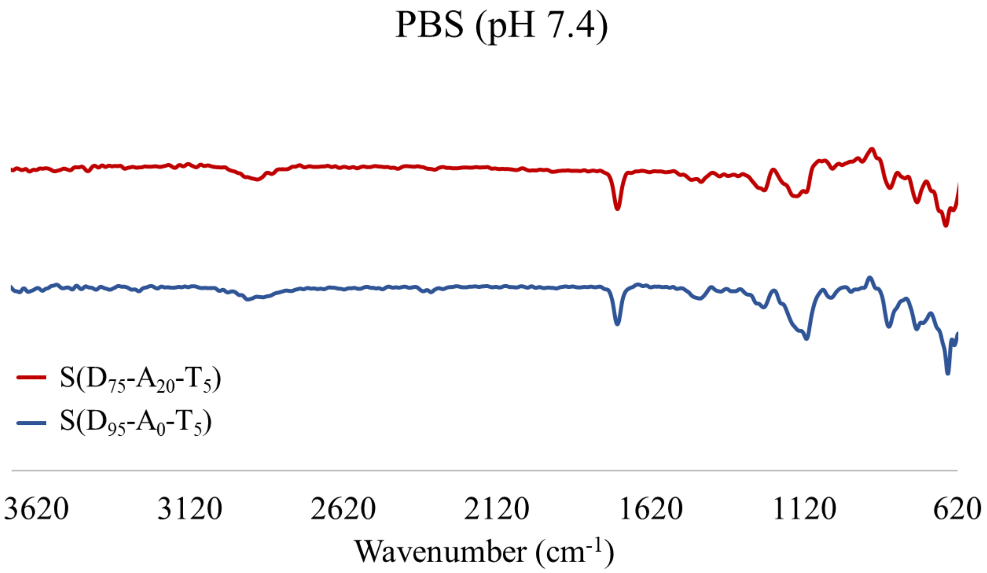


**Figure S4.** FTIR spectra of $S(D_{75}\text{-}A_{20}\text{-}T_5)$ and $S(D_{95}\text{-}A_0\text{-}T_5)$ after incubation with PBS (pH 7.4) at 37°C for 24h.

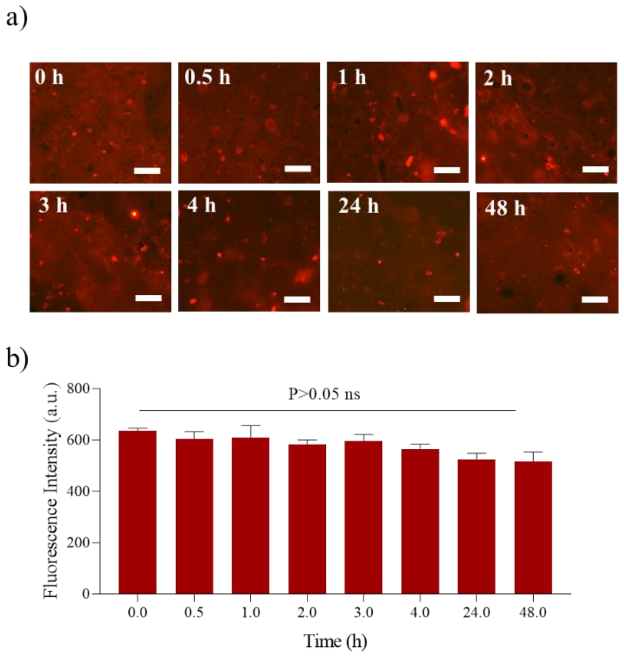


**Figure S5.** The fluorescence images of βCD-grafted lissamine-rhodamine B modified S($D_{75}$-$A_{20}$-$T_5$) surface after incubation at 37 °C in PBS buffer from 0 h to 48 h, b) the fluorescence intensity bar graph of βCD-grafted lissamine-Rhodamine B modified S($D_{75}$-$A_{20}$-$T_5$) surface.

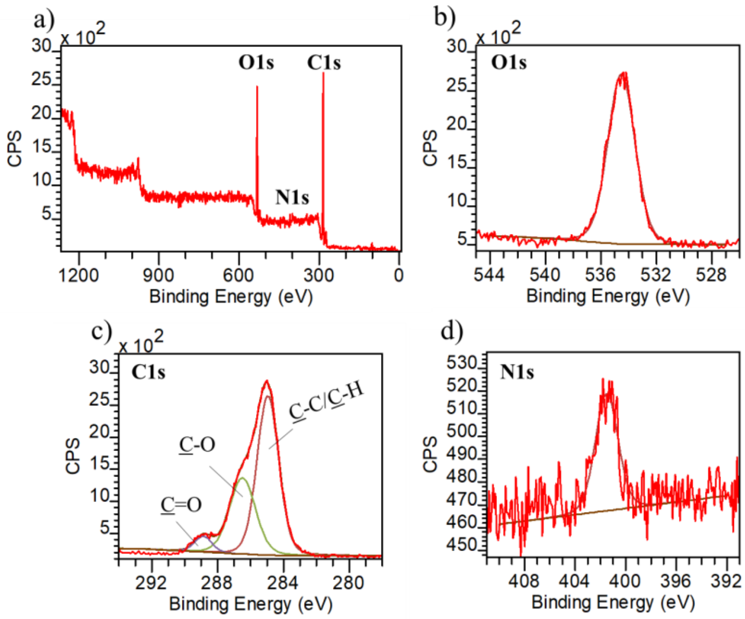


**Figure S6.** (a) The survey scan spectrum, (b) the high-resolution O1s spectra, (c) the high-resolution C1s spectra, and (d) the high-resolution N1s spectra of cRGD-βCD immobilized S($D_{75}$-$A_{20}$-$T_5$).

***Synthesis of βCD-Lissamine Rhodamine B conjugate***

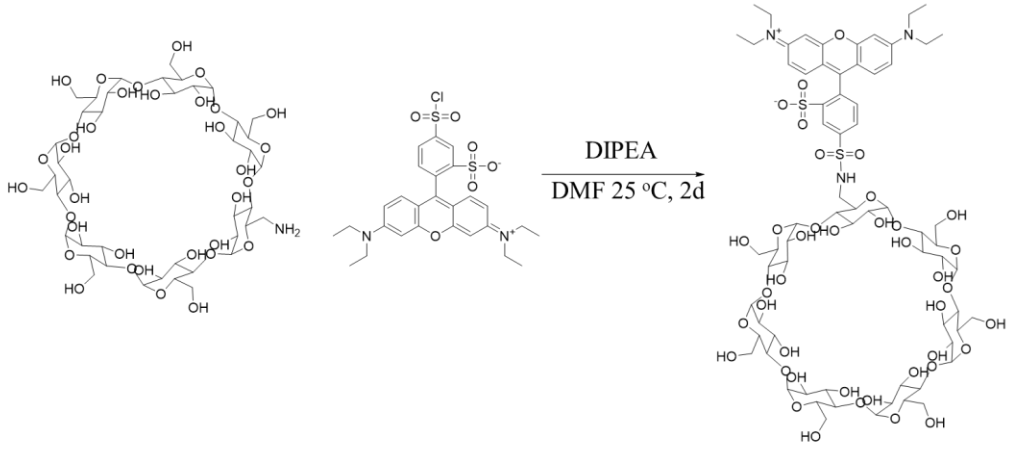


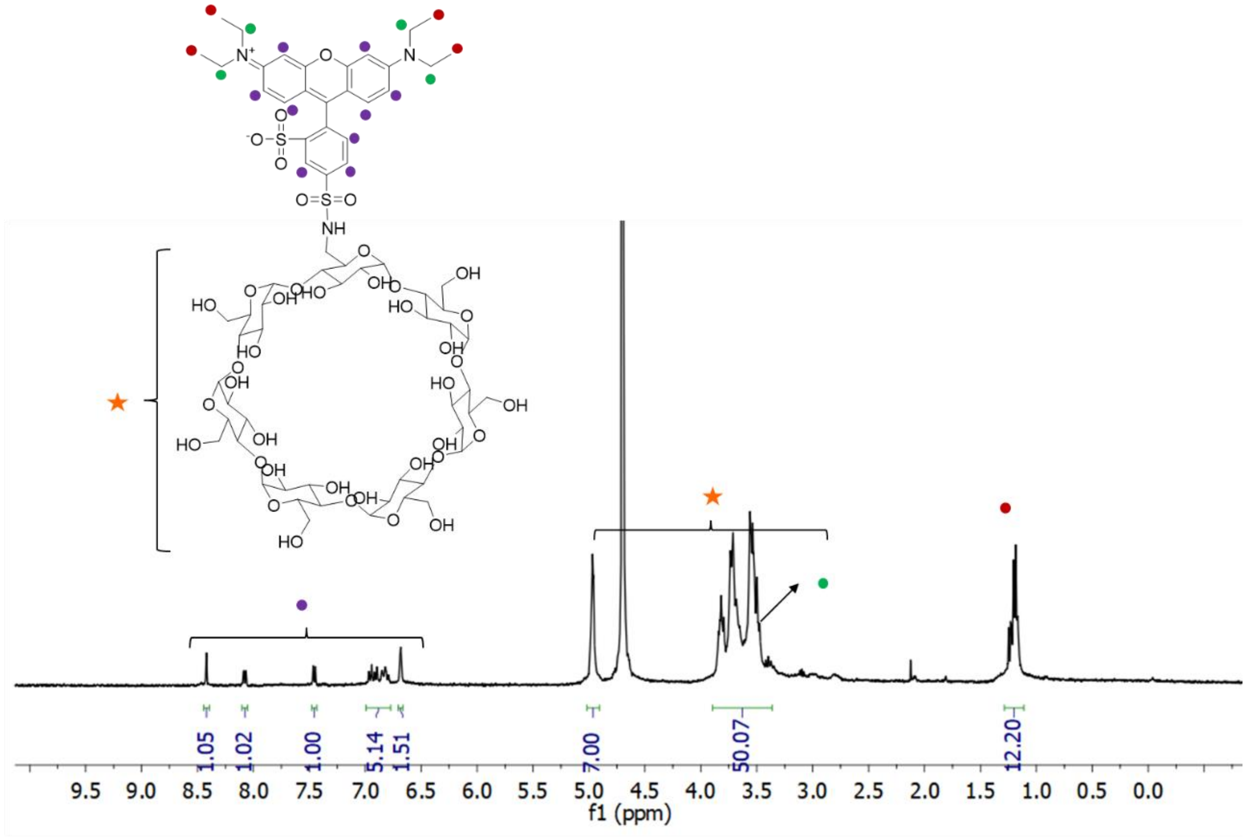


**Figure S7.** $^{1}H$ NMR spectrum of βCD-lissamine rhodamine B conjugate in $D_2O$.

### *Synthesis of mono-mannose conjugated beta cyclodextrin*

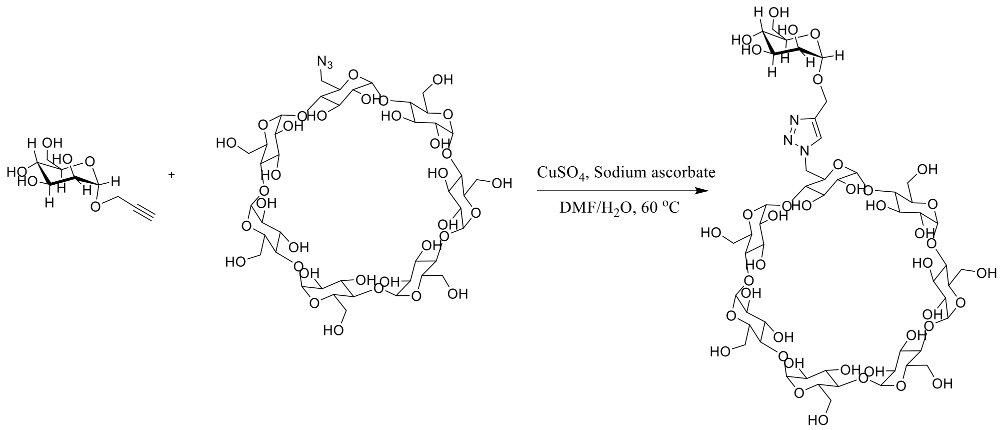


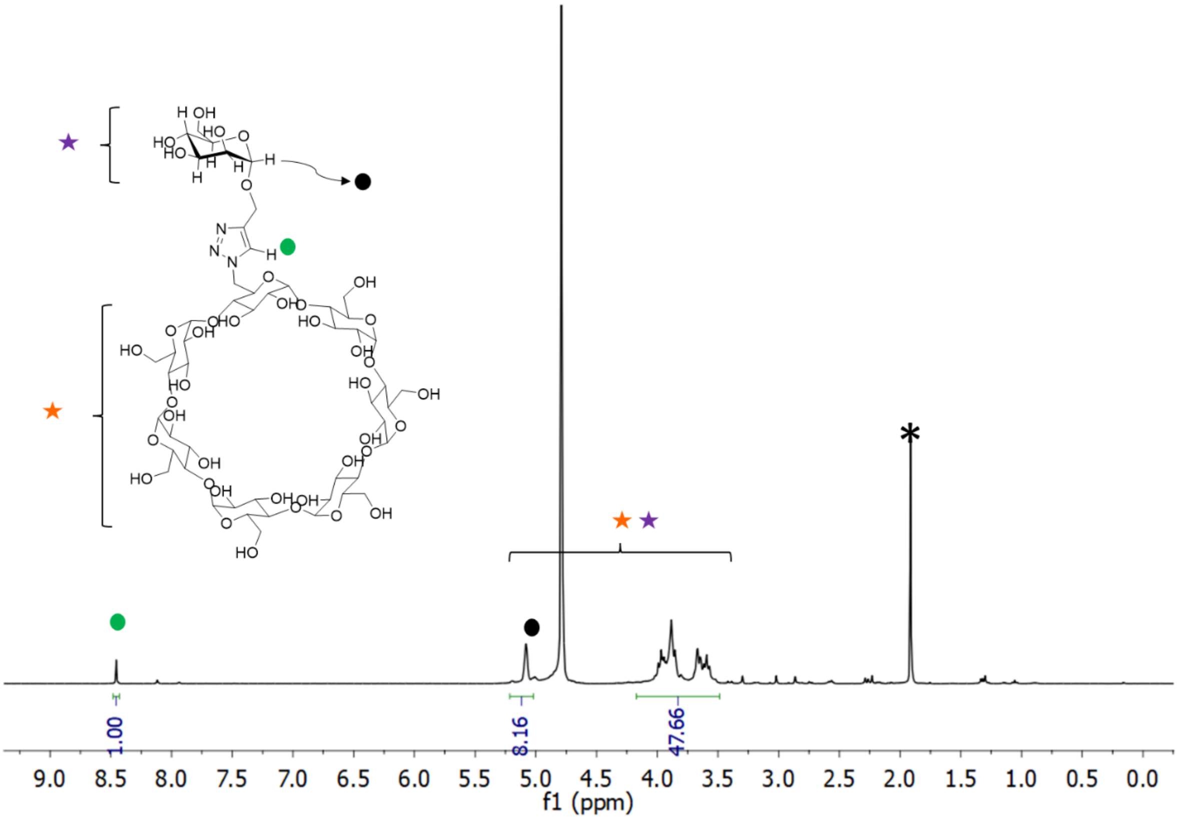


**Figure S8.** $^1$H NMR spectrum of mono-mannose conjugated βCD in $D_2O$.

## *Synthesis of βCD decorated cRGD peptide (cRGD-βCD)*

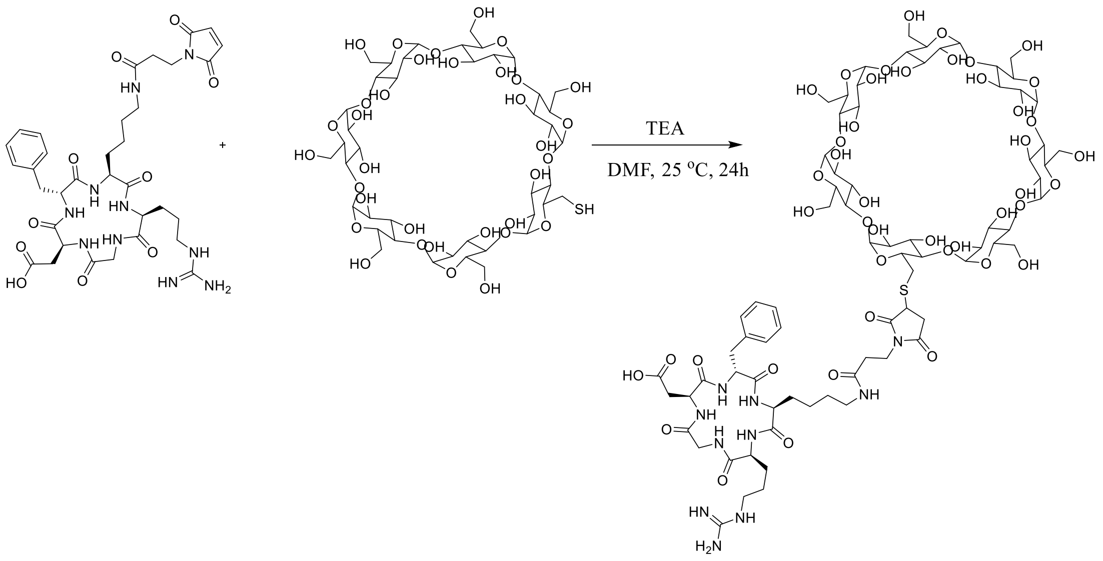


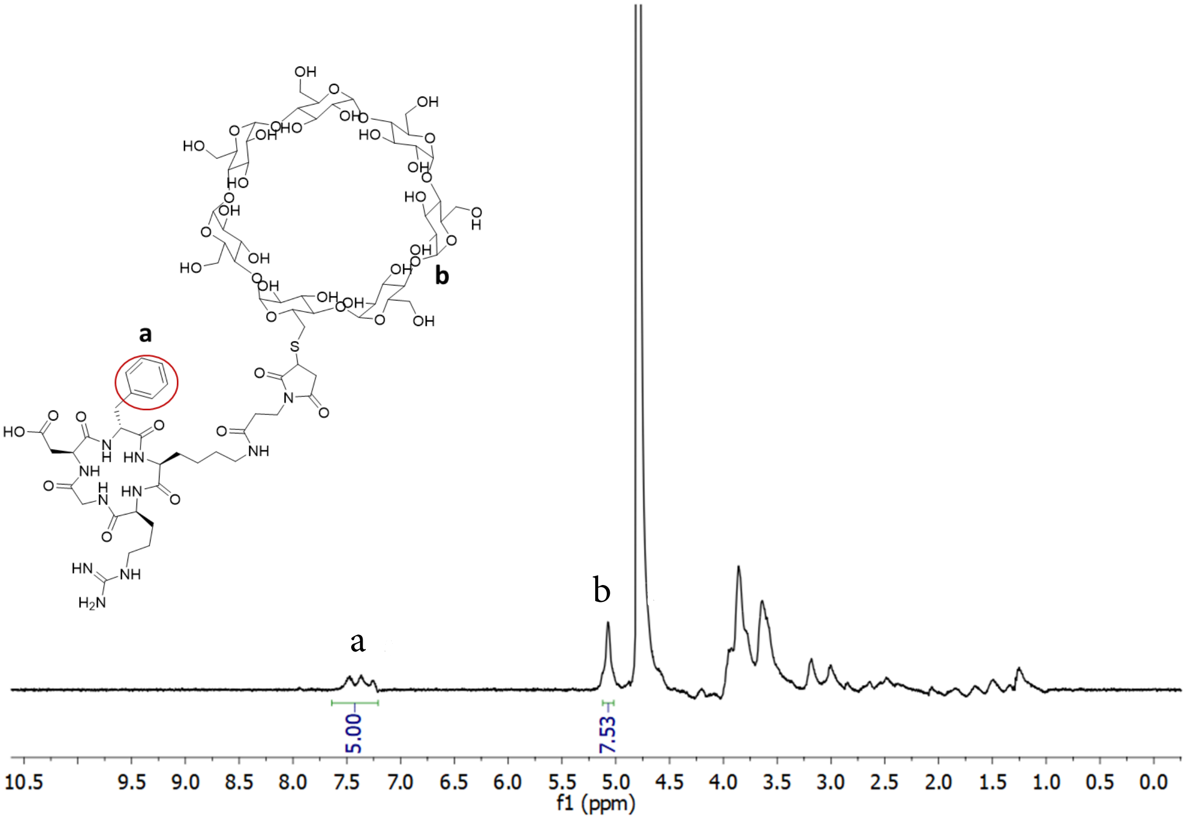


**Figure S9.** $^{1}H$ NMR spectrum of cRGD-βCD in $D_2O$.
